# Calendar

**DOI:** 10.1155/S1463924681000151

**Published:** 1981


### Calendar

**Editor's Note:**
*Organisers of conferences, seminars etc. should send details for inclusion in this calendar as soon as the relevant information is available and not later than three months before the event.*

**Pittsburgh conference**
March 9-13, Atlantic City, USA
*Mrs L. Briggs, Programme Secretary, Pittsburgh Conference, 437 Donald Road, Pittsburgh, Pa 15235, USA*

**Process analytical instrumentation**
March 23-27, Coventry
*Deputy Secretary, Institute of Measurement and Control, 20 Peel Street, London W8*

**2nd International Flow Symposium**
March 23-27, St. Louis, USA
*Dr C. Douglas Hetrick, Director, Membership and Technical Services, ISA Headquarters, PO Box 12277, Research Triangle Park, NC 27709, USA*

**The use of chemical nomenclature**
March 24-26, London
*The Symposium organiser, Laboratory of the Government Chemist, Cornwall House, Stamford Street, London SE1*

**Official methods of analysis and stability testing of new drugs**
March 25-26, Cambridge
*Mr D. Simpson, Analysis for Industry, Factories 2/3, Bosworth House, High Street, Thorpe-le-Soken, Essex CO16 0EA*

**SEMLAB '81**
June 2-5, London
*Industrial Trade Fairs Ltd, Radcliffe House, Blenheim Court, Solihull, West Midlands B91 2BG*

**Chromatography in biochemistry, medicine, and environmental research, 1st international symposium**
June 16-17, Venice
*Alberto Frigerio, Italian Group for Mass Spectrometry in Biochemistry and Medicine, c/o Instituto di Richerche Farmacologiche (Mario Negri), Via Eritrea 62-20157 Milan, Italy*

**Mass spectrometry in biochemistry, medicine, and environmental research, 8th international symposium**
June 18-19, Venice
*Dr Alberto Frigerio, Italian Group for Mass Spectrometry in Biochemistry and Medicine, c/o Instituto di Richerche Farmacologiche (Mario Negri), Via Entrea 62-20157 Milan, Italy*

**Computers in automation and laboratory management; automatic chemical analysis summer school**
July 5-10, Swansea
*Professor D. Betteridge, Chemistry Dept, University College of Swansea, Singleton Park, Swansea*

**Safety of electrical instrumentation in potentially explosive atmospheres, residential course**
July 7-9, Chislehurst
*Conference and courses Unit, Sira Institute Ltd, South Hill, Chislehurst, Kent*

**5th International NMR Meeting**
July 12-16, Exeter
*D. Shaw, Oxford Research Systems, Ferry Hinksey Road, Oxford OX2 ODF*

**Automatica '81**

August 18-22, Oslo

*Information Officer, Norges Varemesse, Drammensvein 154, PO Box 130, Skoyen, Oslo 2, Norway*

**The chemical industry – problems, opportunities, resources. Society of Chemical Industry Centenary Conference**
March 31-April 3, Cambridge
*CENCON, Secretariat, Society of Chemical Industry, 14 Belgrave Square, London SW1X 8PS*

**Laboratory '81 Glasgow**
April 1-2, Glasgow
*Curtis Steadman, 34-36 High Street, Saffron Waldon, Essex.*

**Biotechnology – second European Congress**
April 5-10, Eastbourne
*Secretariat, ECB2, c/o Society of Chemical Industry, 14 Belgrave Square, London SW1X 8PS*

**2nd International Symposium on Flow: its measurement and control in science and industry**
April 6-10, St. Louis, USA
*Symposium Program Committee, Instrument Society of America, 400 Stanwix Street, Pittsburgh, Pa 15222, USA*

**Laboratory '81 Manchester**
April 8-9, Manchester
*Curtis Steadman, 34-36 High Street, Saffron Waldon, Essex.*

**Electroanalysis in clinical, environmental, and pharmaceutical chemistry—international symposium**
April 13-16, Cardiff
*Short Courses Section (Electroanalysis Symposium), UWIST, Cardiff CF1 3NU*

**Automation in the clinical laboratory**
April 19-22, Barcelona
*Dr R. Galimany, Dept de Analisis Clinicos, Seccion de Automatizaction, Cuidad Sanitaria 'Principes de Espana', Hospitalet de Llbrogat, Barcelona, Spain*

**Anglo-Dutch symposium on quantitative organic analysis**
April 23-24, Noordwijkerhout, Holland
*Miss P.E. Hutchinson, Royal Society of Chemistry, Analytical Division, Burlington House, London W1*

**Column liquid chromatography, 5th international symposium**
May 11-15, Avignon, France
*Professor Guiochon, Ecole Polytechnique, Labatoire de Chemie Analytique Physique, Route de Saclay, 91128 Palaiseau Cedix, France*

**Association of Official Analytical Chemists, 6th Annual Spring Workshop**

May 12-14, Ottawa

*Donald L. Grant, Health Protection Branch, Health and Welfare Canada, Tunney's Pasture, Ottawa, Ontario K1A 0LA2, Canada*

**Euroanalysis IV**

August 23-28, Helsinki, Finland

*Euroanalysis IV, Association of Finnish Chemical Societies, Mr. Veikko Velamo, Executive Director, Pohj Hesperiankatu 3B 10, SF-00260 Helsinki 26, Finland*

**Analytical chemistry, 6th Australian symposium**
August 23-28, Canberra, Australia
*Honorary Secretary, Miss B.J. Stevenson, PO Box 1397, Canberra City, ACT 2601, Australia*

**Laboratory '81 London**
September 8-10, London
*Curtis Steadman, 34-36 High Street, Saffron Waldon, Essex*

**Computerised measurement**
September 22-25, Yugoslavia
*Jurema Secretariat, YU-41001 Zagreb, POB 398, Yugoslavia*

**Application of microcomputers in measurement**
September 28-October 3, Yugoslavia
*Jurema Secretariat, YU-41001 Zagreb, POB 398, Yugoslavia*

**ILMAC '81: Laboratory, chemical engineering, measurement, and automation techniques in chemistry exhibition**
September 29-October 2, Basle
*Secretariat Ilmac '81, Postfach, CH-4021 Basle, Switzerland*

**Chemasia '81**
October 21-24, Singapore
*Andrew Dedman, Chemasia '81, Clapp & Poliak Europe Ltd, 232 Acton Lane, London W4 5DL*

